# Total knee arthroplasty in dialysis patients: a national in-patient sample-based study of perioperative complications

**DOI:** 10.1186/s43019-023-00196-0

**Published:** 2023-08-02

**Authors:** Nikit Venishetty, Dane K. Wukich, Jack Beale, J. Riley Martinez, Michel Toutoungy, Varatharaj Mounasamy, Senthil Sambandam

**Affiliations:** 1 Paul L. Foster School of Medicine, Texas Tech University Health Sciences Center, El Paso, TX USA; 2 Department of Orthopedics, University of Texas Southwestern, Dallas VAMC, Dallas, TX USA

**Keywords:** Total knee arthroplasty, Dialysis, Length of stay, Postoperative complications, Cost of care, Chronic kidney disease

## Abstract

**Background:**

Chronic kidney disease (CKD) is a growing disease that affects millions of people in the USA every year. Many CKD patients progress to end-stage renal disease (ESRD), necessitating the use of hemodialysis to alleviate symptoms and manage kidney function. Furthermore, many of these patients have lower bone quality and experience more postoperative complications. However, there is currently limited information on hospitalization information and perioperative complications in this population following procedures such as total knee arthroplasty (TKA). The purpose of this study was to assess the patient characteristics, demographics, and prevalence of postoperative problems among dialysis patients who received TKA.

**Methods:**

In this retrospective study, we used the Nationwide Inpatient Sample (NIS) data from 2016 to 2019 to analyze the incidence of perioperative complications, length of stay (LOS), and the cost of care (COC) among patients undergoing TKA who were categorized as dialysis patients, compared with those who were not. Propensity matching was conducted to consider associated factors that may influence perioperative complications.

**Results:**

From 2016 to 2019, 558,371 patients underwent TKAs, according to the National In-Sample (NIS) database. Of those, 418 patients (0.1%) were in the dialysis group, while the remaining 557,953 patients were included in the control group. The mean age of the dialysis group was 65.4 ± 9.8 years, and the mean age in the control group was 66.7 ± 9.5 years (*p* = 0.006). After propensity matching, dialysis group patients had a higher risk of receiving blood transfusions [odds ratio (OR): 2; 95% confidence interval (CI): 1.2, 3.4] and a significantly larger COC in comparison to those in the control group (91,434.3 USD versus 71,943.6 USD, *p* < 0.001). In addition, dialysis patients had significantly higher discharges to another facility, as compared with the control group patients.

**Conclusions:**

The dialysis group had a significantly higher cost of care, higher rates of requiring blood transfusion, and more cases of being discharged to another facility than non-dialysis patients. This data will help providers make informed decisions about patient care and resource allocation for dialysis patients undergoing TKA.

## Introduction

According to the Centers for Disease Control and Prevention (CDC), an estimated 10% of the population worldwide is affected by chronic kidney disease (CKD), with one in seven people in the USA affected [1, 2]. In 2018 alone, approximately 780,000 Americans had renal failure requiring dialysis or a kidney transplant for survival [2]. More than 70% of chronic kidney failure patients receive dialysis; however, these patients are prone to advanced osteoarthritis and osteonecrosis of the knee, which often necessitates total knee arthroplasty [3].

Total knee arthroplasty (TKA), a surgical intervention where the articular surface of the knee joint is replaced with prosthetic components, accounted for nearly 686,000 procedures in 2010 in the USA [4, 5]. As dialysis patients live longer, they have an increasing need for TKA to improve joint function, relieve pain, and improve quality of life. End-stage renal disease (ESRD) patients often have comorbid cardiovascular disease (e.g., coronary artery disease, impaired myocardial function, and carotid stenosis) that is associated with higher rates of perioperative complications [3, 6]. Previous studies have shown that dialysis patients have a higher risk of complications following TKA [3, 6]. However, these studies have limited information on a variety of postoperative complications and do not take into account a large sample size.

While numerous patients on dialysis require procedures such as TKA and total hip arthroplasty, there is little information on the perioperative complications that occur within this patient population. In this study, the Nationwide Inpatient Sample (NIS) Database was queried to assess the patient characteristics, demographics, and prevalence of postoperative problems among dialysis patients who received TKA. We hypothesize that dialysis patients will have higher rates of perioperative complications, longer hospitalizations, and higher costs associated with their care in comparison with non-dialysis patients.

## Methods

### Database description

The NIS, which includes information on more than 7 million hospital stays, is the largest publicly accessible all-payer inpatient care database in the USA. This database was created through a federal–state–industry partnership supported by the Agency for Healthcare Research and Quality. It is a member of a family of databases and software tools created for the Healthcare Cost and Utilization Project (HCUP; AHRQ). The NIS’s large sample size makes it possible to create regional and national estimates and allows for the analysis of groups with unique characteristics, such as dialysis patients undergoing TKA. The information is based on a quality assessment analysis by an independent contractor that compares data points with accepted normative values and examines 20% of the hospitals in the USA. The NIS database contains data on patient demographics, length of stay (LOS), payment source, hospital charges, discharge status, comorbidities, and several other variables. For the 2016–2019 revision, the International Classification of Diseases (ICDs), Tenth Revision, and Clinical Modification/Procedure Coding System is employed.

### Data acquisition

Our Institutional Review Board (IRB) determined that this study was exempt from clearance because the data were de-identified and readily available to the public. All patients with ICD-10, Tenth Revision, and Clinical Modification/Procedure Coding System (CMP) code for TKA were included in this study. Patients were then classified into two groups: namely dialysis patients and patients who did not undergo dialysis (control). Data was extracted from 2016 to 2019, and the ICD codes used for this study are described in Appendix A.

Age, sex, ethnicity, and the presence of obesity were all considered in the analysis of demographic factors. Additionally, comorbidities and postoperative complications [postoperative anemia, hypotension, acute renal failure (ARF), deep vein thrombosis (DVT), and pulmonary embolism (PE)] were also included. Our analysis considered local issues such as periprosthetic infections (PPIs), prosthetic dislocations, and periprosthetic fractures in addition to systemic medical consequences such as myocardial infarction (MI) and pneumonia. Moreover, the patients’ post-hospitalization dispositions [short-term hospital, home/routine, alternative facility, home healthcare (HHC), and left against medical device (LAMA)] were examined. Finally, information was gathered for each of these patients regarding the length of time spent in the hospital or the procedures they underwent (total length of stay). All preoperative variables and postoperative complications were based on ICD codes described in Appendix A.

### Statistical analysis

SPSS version 27.0 was used for all statistical analyses (IBM; Armonk, NY, USA). Demographic information about patients was initially compiled using descriptive statistics. We carried out a matched and unmatched analysis. Using the preoperative variables, a 1:1 propensity matching algorithm was run. Propensity matching was conducted for age, sex, race, obesity, tobacco-use, diabetes with complications, and diabetes without complications. When examining numerical variables, *t*-tests were employed. Binomial variables were examined using chi-squared analysis. When the incidence values were less than 5, Fisher exact tests were applied. For all tests, a *p*-value of 0.05 or lower was regarded as statistically significant. As a ratio of the incidence in the dialysis group to the incidence in the control group, odds ratios, and their accompanying 95% confidence intervals (CIs) for surgical outcomes and complications were calculated.

## Results

A total of 558,371 patients were identified in the NIS database who underwent TKA between 2016 and 2019. Of this cohort, 418 patients (0.1%) were in the dialysis group, while the remaining 557,953 patients were included in the control group. Baseline characteristics between these two groups were analyzed and compared. In unmatched analysis, the mean age of the dialysis group was 65.4 ± 9.8 years, and the mean age in the control group was 66.7 ± 9.5 years (*p* = 0.006). In addition, the control group had significantly higher rates of obesity (30.9%) versus the dialysis group (22%, *p* < 0.001). Additionally, in looking at patient ethnicity, for both groups, there was a greater proportion of Caucasians in comparison to other races such as African American, Asian or Pacific Islander, and Native American (Table 1). 1:1 propensity matching was conducted for age, sex, obesity, and race and diabetes with and without complications, and after matching there were no significant differences between the two groups (Table 2).

**Table 1:** Unmatched analysis—patient demographic characteristics of dialysis patients and the control patients

	Dialysis group (418)	Control group (557,953)	Significance
Age in years	65.4 (SD = 9.8)	66.7 (SD = 9.5)	**0.006**
Female	195 (46.7%)	343,225 (61.5%)	** < 0.001**
Obesity	92 (22%)	172,569 (30.9%)	** < 0.001**
Race			
Caucasian	184 (44%)	435,033 (77.9%)	** < 0.001**
African American	145 (34.7%)	44,694 (8%)	** < 0.001**
Hispanic	50 (11.9%)	33,544 (6%)	** < 0.001**
Asian	11 (2.6%)	8240 (1.6%)	** < 0.001**
Native American	1.9%	2523 (0.5%)	** < 0.001**
Other	11 (2.6%)	4835 (0.9%)	** < 0.001**

Bolded values indicate statistical significance

Numbers between 1 and 10 were not reported as per healthcare cost and utilization project (HUCP) data agreement

SD, standard deviation

**Table 2:** Matched analysis—patient demographic characteristics of dialysis patients and the control patients

	Dialysis group (418)	Control group (409)	Significance
Age in years	65.4 (SD = 9.8)	64.7 (SD = 10.5)	0.3
Female	195 (46.7%)	189 (46.2%)	0.5
Obesity	92 (22%)	90 (22%)	0.9
Race			
Caucasian	184 (44%)	184 (44%)	0.5
African American	145 (34.6%)	145 (35.4%)	0.5
Hispanic	50 (11.9%)	50 (12.2%)	0.5
Asian	11 (2.6%)	11 (2.7%)	0.5
Native American	1.9%	1.9%	0.5
Other	11 (2.6%)	11 (2.7%)	0.5

Numbers between 1 and 10 were not reported as per healthcare cost and utilization project (HUCP) data agreement

SD, standard deviation

### Patient admission characteristics

In the unmatched analysis, dialysis group patients had significantly greater the cost of care (COC) and a longer LOS than patients in the control group. In addition, dialysis patients had higher rates of tobacco-related disorders, and diabetes with complications (Table 3). Dialysis patients were significantly more likely to be discharged to another facility rather than home. In comparison to dialysis patients, the control group used home care more often (Table 3). After 1:1 propensity matching, patients in the dialysis group (91,434.3 USD) had a significantly larger expenditure in comparison to those in the control group (71,943.6 USD, *p* < 0.001). Dialysis patients were more likely discharged to another facility than home, and the control group used HHC more often (Table 4). Nearly 40% of dialysis patients needed discharge to another facility. Moreover, the HHC rates were much lower for dialysis patients (37.4% versus 57.5%, *p* < 0.001). In addition, 34,204 patients that were diagnosed with CKD did not undergo dialysis. After matched analysis, only 20 patients were diagnosed with CKD and did not undergo dialysis.

**Table 3:** Unmatched analysis—admission characteristics of dialysis patients and controls

Variable	Dialysis group (*n* = 418)	Control group (*n* = 557,953)	Significance	Odds ratio 95% CI
Age in years at admission (years)	65.4 (SD = 9.8)	66.7 (SD = 9.5)	** < 0.001**	(5.3, 5.5)
Length of stay (days)	3.8 (SD = 2.8)	2.3 (SD = 1.9)	** < 0.001**	(0.4, 0.4)
Total charges (USD)	91,434.3 (SD = 69,572.1)	64,791.1 (SD = 45,806.7)	** < 0.001**	(3985.9, 4989.3)
Elective versus non-elective admission	535,229 (96.1%)	380 (90.9%)	** < 0.001**	(0.3, 0.6)
Tobacco-related disorder	48 (11.5%)	88,321 (15.8%)	**0.01**	
Diabetes without complications	14 (3.3%)	82,450 (14.8%)	** < 0.001**	
Diabetes with complications	0.2%	1403 (0.3%)	** < 0.001**	
Patient disposition				
Home/routine	98 (23.5%)	207,368 (37.2%)	** < 0.001**	
Short-term hospital	0.7%	1315 (0.2%)	** < 0.001**	
Another type of facility	159 (38%)	103,390 (18.5%)	** < 0.001**	
Home healthcare (HHC)	156 (37.3%)	245,044 (43.9%)	** < 0.001**	
Left against medical device (LAMA)	0.2%	329 (0.1%)	** < 0.001**	
Died	0.2%	197 (0.04%)	** < 0.001**	

Bolded values indicate statistical significance

Numbers between 1 and 10 were not reported as per healthcare cost and utilization project (HUCP) data agreement

SD, standard deviation

**Table 4:** Matched analysis—admission characteristics of dialysis patients and controls

Variable	Dialysis group (*n* = 418)	Control group (*n* = 409)	Significance	Odds ratio 95% CI
Age in years at admission (years)	65.4 (SD = 9.8)	64.7 (SD = 10.5)	0.3	(−0.7, 2.1)
Length of stay (days)	3.8 (SD = 2.8)	3.8 (SD = 1.9)	0.9	(−0.6, 0.6)
Total charges (USD)	91,434.3 (SD = 69,572.1)	71,943.6 (SD = 56,806.7)	** < 0.001**	(7390.4, 31,591.2)
Tobacco-related disorder	48 (11.5%)	61 (14.9%)	0.1	
Diabetes without complications	14 (3.3%)	14 (3.4%)	0.5	
Diabetes with complications	0.2%	0.2%	0.7	
Patient disposition				
Home/routine	98 (23.5%)	44 (10.8%)	** < 0.001**	
Short-term hospital	0.7%	1%	** < 0.001**	
Another type of facility	159 (38%)	123 (30%)	** < 0.001**	
Home healthcare (HHC)	156 (37.4%)	235 (57.5%)	** < 0.001**	
Left against medical device (LAMA)	0.2%	0.2%	** < 0.001**	
Died	0.2%	0.5%	0.5	

Bolded values indicate statistical significance

Numbers between 1 and 10 were not reported as per healthcare cost and utilization project (HUCP) data agreement

SD, standard deviation

### Unmatched complications and adverse events during hospital admission

Compared with the control group, dialysis patients are more likely to die during hospitalization (OR: 6.8; 95% CI: 1, 48.5). Dialysis patients had significantly higher rates of blood transfusion (OR: 8.1, 95% CI: 5.9, 11), blood loss anemia (OR: 1.5; 95% CI: 1.2, 1.9) periprosthetic fracture (OR: 2.9; 95% CI: 1.2, 6.9), and periprosthetic infection (OR: 5.6; 95% CI: 3.7, 8.5) than patients in the control group. There were no statistically significant differences between the dialysis group and the control group for MI, pneumonia, PE, DVT, and periprosthetic mechanical complications (Table 5).

**Table 5:** Unmatched analysis—in-hospital complications of dialysis patients and the control group

Post-operative variables	Dialysis group (418)	Control group (557,953)	Odds ratio (dialysis/control group)	Odds ratio 95% confidence interval	Significance
Died during hospitalization	0.2%	197 (0.03%)	6.8	(1, 48.5)	** < 0.001**
Acute renal failure	0.7%	11,082 (2%)	0.4	(0.1, 1.1)	0.1
Myocardial infarction	0	109 (0.01%)	0.99	(0.99, 0.99)	0.8
Blood loss anemia	90 (21.5%)	85,467 (15.3%)	1.5	(1.2, 1.9)	** < 0.001**
Pneumonia	0.5%	1086 (0.2%)	2.5	(0.6, 9.9)	0.19
Blood transfusion	45 (10.8%)	8209 (1.5%)	8.1	(5.9, 11)	** < 0.001**
Pulmonary embolism	0	1238 (0.2%)	0.99	(0.99, 0.99)	0.34
Deep vein thrombosis	0.7%	1260 (0.2%)	2.1	(0.5, 8.5)	0.28
Periprosthetic fracture	5 (2%)	2358 (0.4%)	2.9	(1.2, 6.9)	**0.02**
Periprosthetic dislocation	2%	4257 (0.8%)	1.6	(0.7, 3.8)	0.31
Periprosthetic infection	23 (5.5%)	5779 (1%)	5.6	(3.7, 8.5)	** < 0.001**
Periprosthetic mechanical complication	2%	4499 (0.8%)	1.5	(0.6, 3.6)	0.37

Bolded values indicate statistical significance

Numbers between 1 and 10 were not reported as per healthcare cost and utilization project (HUCP) data agreement

### Matched complications and adverse events during hospital analysis

The 1:1 propensity match algorithm yielded 409 patients in the control group and 418 patients in the dialysis group. The incidence of blood transfusions was much greater in the dialysis group, 10.8%, than in the control group, 5.6% (*p* = 0.005). The significance of other postoperative complications attenuated and demonstrated no significant differences among the two groups in the matched analysis (Table 6).

**Table 6:** Matched analysis—in-hospital complications of dialysis patients and the control group

Post-operative variables	Dialysis group (418)	Control group (409)	Odds ratio (dialysis/control group)	Odds ratio 95% confidence interval	Significance
Died during hospitalization	0.2%	0.5%	0.5	(0.04, 5.4)	0.5
Acute renal failure	0.7%	17 (4.1%)	0.2	(0.1, 0.6)	** < 0.001**
Myocardial infarction	0	0.2%	0.99	(0.5, 0.5)	0.5
Blood loss anemia	90 (21.5%)	73 (17.8%)	0.99	(0.1, 6.9)	0.1
Pneumonia	0.5%	0.5%	2.5	(0.6, 9.9)	0.7
Blood transfusion	45 (10.8%)	23 (5.6%)	2	(1.2, 3.4)	**0.005**
Pulmonary embolism	0	0.2%	0.99	(0.5, 0.5)	0.5
Deep vein thrombosis	0.5%	0.5%	0.7	(0.1, 3.9)	0.5
Periprosthetic fracture	2%	1.2%	0.98	(0.3, 3.4)	0.6
Periprosthetic dislocation	2%	1.2%	1.6	(0.3, 4.6)	0.5
Periprosthetic infection	23 (5.5%)	23 (5.6%)	1.3	(0.7, 2.5)	0.2
Periprosthetic mechanical complication	2%	1.2%	0.98	(0.3, 3.4)	0.6

Bolded values indicate statistical significance

Numbers between 1 and 10 were not reported as per healthcare cost and utilization project (HUCP) data agreement

## Discussion

With the increasing prevalence of chronic kidney failure and more patients requiring dialysis, there is likely to be an increase in the rates of TKA in this patient population [3]. Consistent with our study, previous studies have also found that dialysis patients have a higher risk of complications following TKA [3, 6–8].

In unadjusted analyses, dialysis patients were more likely to have higher rates of periprosthetic fracture and periprosthetic infection. After propensity matching, we found that there were no significant differences in periprosthetic infections between dialysis and non-dialysis patients. This similarity after matching may be explained, as propensity matching variables such as diabetes mellitus, tobacco use, age, sex, obesity, and race can be associated with increased infections in dialysis patients [9–11]. Thus, after matching, the control group may have a higher incidence of periprosthetic infection. We included periprosthetic infections, fractures, and dislocations as postoperative complications in this study since it is uncommon in this population, and it is difficult to find these complications in single-institutional studies.

Most dialysis patients who underwent TKA were Caucasian, independent of age. This is consistent with the study by Ponnusamy et al. [7], who reported that the majority of their dialysis patients were Caucasian (41.7%), and the mean age of the dialysis group was significantly higher than that of the control group. According to the CDC’s CKD report for 2021, patients ages 65 years and older have the highest prevalence of CKD (38%), followed by those ages 45–64 years (12%) and ages 18–44 years (6%) [2]. However, we found that the non-dialysis group had a significantly greater age than the control group, but this difference attenuated when propensity matching was performed.

Dialysis patients had a significantly longer hospital LOS, which contributes to higher hospital costs. A retrospective study from the National Health Insurance Research Database in Taiwan reported that patients with ESRD who underwent TKA had a significantly longer length of hospital stays (8.2 ± 1.3 days) than those in the non-ESRD group (7.5 ± 0.8 days) [12]. Perioperative complications contributed to a longer stay (12.1 ± 3.7 days). While this does not directly compare dialysis patients to non-dialysis patients, more than 71% of ESRD patients use dialysis to manage their symptoms [2, 3, 7]. Additionally, the study in Taiwan found that ESRD patients had a significantly higher cost of expenses (4210.1 ± 382.8 USD) than those without ESRD (3791.2 ± 307.3 USD). This cost increased in both groups as complications increased [12].

Moreover, we found that dialysis patients in this study had a higher incidence of discharges to another facility. Patterson et al. [13] similarly demonstrated that the presence of dialysis has also been linked to broader risks of occurrence of any adverse events, need for critical care, and facility release in patients undergoing joint arthroplasty. This may be attributed to the higher rates of postoperative complications found among dialysis patients [7, 13].

Regarding perioperative complications, we found that dialysis patients had significantly higher blood transfusion rates. Dialysis patients are more prone to blood loss and anemia necessitating blood transfusions due to multiple factors, including preoperative anemia of chronic disease, low erythropoietin levels, and a higher perioperative bleeding risk due to the uremic effect on platelets [14]. This is consistent with a study conducted by Ottesen et al. [15], as they reported that dialysis patients who underwent TKA had a higher likelihood of developing a serious adverse event than patients not on dialysis. These two variables may be related because CKD patients typically have chronic anemia (erythropoietin is synthesized in the kidney) and a higher risk of bleeding due to the uremic effects on platelet count [16–18]. Ponnuswammy et al. [7] demonstrated that dialysis patients had significantly higher rates of transfusion than non-dialysis-dependent patients [7]. Although the blood transfusion rates remained significantly different after matching, blood loss anemia was found to remain similar between both groups. This may be explained by the association of propensity-matching comorbidities and blood loss anemia after TKA. In addition, poor tolerance for volume load and loss may require surgeons to conduct allogenic blood transfusions more frequently.

Another potential finding of this study is the presence of health disparities of dialysis patients who are offered primary TKA. According to the 2020 Census Bureau’s population estimate of the racial and ethnic makeup of the USA, Caucasians (61.9%) made up the majority of the population, followed by Hispanic/Latino (18.7%), African American (12.4%), Asian (6%), Native American (1.1%), and Pacific Islander (0.2%) patients [19]. We found that the control group had a large disparity in TKA patients among ethnic groups, where Caucasians predominated (77.9%), followed by African American (8%), Hispanic (6%), Asian or Pacific Islander (1.5%) and Native American (0.5%) patients. While we primarily looked at mortality, length of stay, and perioperative complications in dialysis patients undergoing TKA, the disparity present within our analysis sheds light on an important topic that merits further discussion.

## Limitations

We recognize that the NIS, the largest database currently accessible involving all players, may be prone to deficiencies in variables such as long-term outcomes of TKA in dialysis patients (e.g., information regarding 30-day and 90-day post-operative mortality). Despite these limitations, a study from 2013 demonstrated that administrative registries of total joint arthroplasty patients that evaluated comorbidities and complications correlated well with institutional clinical series. Bozic et al. demonstrated that comorbidities and complications after total joint arthroplasty had a specificity of more than 92%, but the sensitivity ranged from 29% to 100% [20]. The authors found a high degree of concordance between the clinical and administrative records for certain comorbidities (diabetes, chronic lung disease, and coronary artery disease) and complications (postoperative MI) in patients undergoing total joint arthroplasty but only moderate concordance for other comorbidities (congestive heart failure, obesity, prior myocardial infarction, peripheral artery disease, and a history of thromboembolism) and complications (DVT/PE, bleeding complications, and prosthetic-related complications). Even though the information coded in NIS is reasonably accurate (specificity > 92%), this information can be lacking.

In addition, using ICD codes to generate comparisons about patient samples may limit the validity of the results. This may not capture the patient population’s full characteristics, and a comparison of exact data from institutions would be more accurate. We also found that the control group had a higher rate of acute renal failure in comparison to the dialysis group, and this is likely due to the unavailability of dialysis treatment for the control group. Moreover, matched analysis of race, age, sex, obesity, tobacco use, and diabetes (with or without complications) attenuated the significance of several postoperative complications. Lastly, other confounding variables that may have impacted the perioperative complications were not chosen due to the limited number of variables available in the NIS database. Thus, we felt using some of the most common hospital characteristics encountered when assessing propensity matching was appropriate. However, using a large sample size, this study provides important information regarding the characteristics of dialysis patients undergoing TKA.

## Conclusions

The dialysis group had a significantly higher cost of care, higher rates of requiring blood transfusion, and more cases and more cases of being discharged to another facility than non-dialysis patients. This study provides information on the perioperative results of primary TKA in dialysis patients to providers, healthcare organizations, and clinicians. When assessing hospital expenditures for dialysis patients, it is important to consider the higher rate of perioperative problems in this patient group. This data will help providers make informed decisions about patient care and resource utilization for dialysis patients undergoing TKA.

## Data Availability

The datasets used and/or analyzed during the current study are available from the corresponding author on reasonable request.

## Appendix A: ICD codes

**Table:**

Obese codes	Morbidly obese codes	Comorbidities codes	Medical complications codes	Surgical complications codes
E660 E6601 E6609 E661 E662 E668 E669 Z6830 Z6831 Z6832 Z6833 Z6834 Z6835 Z6836 Z6837 Z6838 Z6839	Z6841 Z6842 Z6843 Z6844 Z6845	Diabetes without complications E119 Diabetes with complications E1169 Tobacco-related disorder Z87891	Acute renal failure N170, N171, N172, N178, N179 Myocardial infarction I2101, I2102, I2111, I2113, I12114, I12119, I2121, I12129, I21A1 Blood loss anemia D62 Pneumonia J189, J159, J22 Blood transfusion 30233N1 Pulmonary embolism I2602, I2609, I2692, I2699 DVT I82401, I82402, I82403, I82409, I82411, I82412, I82413, I82419, I82421, I82422, I82423, I82429, I82431, I82432, I82433, I82439, I82441, I82442, I82443, I82449, I82491, I82492, I82493, I82499, I824Y1, I824Y2, I824Y3, I824Y9, I824Z1, I824Z2, I824Z3, I824Z4	Periprosthetic fracture T84010A, T84011A, T84012A, T84013A, T84018A, T84019A, M9665, M96661, M96662, M96669, M96671, M96672, M96679, M9669, M9701XA, M9702XA, M9711XA, M9712XA Periprosthetic dislocation T84020A, T84021A, T84022A, T84023A, T84028A, T84029A Periprosthetic mechanical complications T84090A, T84091A, T84092A, T84093A, T84098A, T84099A Periprosthetic infection T8450XA, T8451XA, T8452XA, T8453XA, T8454XA, T8459XA Superficial SSI T8141XA Deep SSI T8142XA Wound dehiscence T8130XA, T8131XA, T8132XA
